# Association of plasma C-reactive protein level with the prevalence of colorectal adenoma: the Colorectal Adenoma Study in Tokyo

**DOI:** 10.1038/s41598-017-04780-9

**Published:** 2017-06-30

**Authors:** Nano Kigawa, Sanjeev Budhathoki, Taiki Yamaji, Motoki Iwasaki, Manami Inoue, Shoichiro Tsugane

**Affiliations:** 1 Department of Global Health Policy, Graduate School of Medicine, The University of Tokyo, Tokyo, Japan; 2 Epidemiology and Prevention Group, Center for Public Health Sciences, National Cancer Center, Tokyo, Japan; 3 AXA Department of Health and Human Security, Graduate School of Medicine, The University of Tokyo, Tokyo, Japan

## Abstract

Epidemiologic studies have identified a positive association between obesity and colorectal neoplasia. Adiposity induces systemic low-grade inflammation, which is commonly assessed with a sensitive biomarker, C-reactive protein (CRP). To understand the molecular mechanisms of obesity in the etiology of colorectal neoplasia, the present study was conducted in 782 adenoma cases and 738 controls who underwent total colonoscopy, and their plasma CRP level was evaluated in relation to colorectal adenoma prevalence. A logistic regression model was used to compute odds ratios (OR) and 95% confidence intervals (CI) of adenoma according to quartile of plasma CRP. Plasma CRP level was positively associated with higher adenoma prevalence in all subjects (OR 1.30; 95% CI 0.94–1.79 for the highest versus lowest quartile; *P* trend = 0.031). Further analysis by adenoma size and number revealed a pronounced association with a larger size (≥5 mm) and multiple numbers (≥2). These positive associations were reduced to non-significance following further adjustment for body mass index, and OR for the highest versus lowest quartile of plasma CRP became 1.12 (95% CI 0.80–1.56; *P* trend = 0.25) in all subjects. In conclusion, this study suggests that obesity-related systemic low-grade inflammation may play an important role in the early stages of colorectal carcinogenesis.

## Introduction

Overweight and obesity are consistently associated with an increased risk of colorectal cancer and adenoma^1–4^. Epidemiological evidence indicates that the risk of colorectal cancer is about 19% higher in obese people than in those of normal weight, and increases about 4–10% per 2-kg/m^2^ rise in body mass index (BMI)^5^. Similarly, for colorectal adenoma, a meta-analysis of 36 epidemiological studies which included a total of 29,860 cases found a 19% increase in adenoma risk associated with a 5-kg/m^2^ increase in BMI^6^. However, the biological link underlying this association has not been fully elucidated.

Adipose tissue, although previously considered to be a passive storage organ, is now known to secrete a variety of inflammatory cytokines, including tumor necrosis factor-α (TNF-α) and interleukin-6 (IL-6)^7–9^. Excessive secretion of these cytokines from hypertrophic adipocytes following obesity is postulated to induce and maintain a systemic, low-grade inflammatory state, which may contribute to the initiation and progression of carcinogenesis^7–9^. In fact, experimental studies have consistently shown that oxidative stress due to systemic inflammation likely inhibits the function of tumor suppressor gene *APC* (adenomatous polyposis coli) and *p*53, which are closely related to the development of colorectal adenoma and progression to colorectal cancer, respectively^10^.

C-reactive protein (CRP) is readily produced in response to inflammatory cytokines by hepatocytes, and accordingly serves as a useful biomarker of systemic inflammatory states. The importance of CRP in this role is further strengthened by its much higher circulating concentrations than individual inflammatory cytokines. Nonetheless, epidemiological studies evaluating the association between circulating CRP level and colorectal adenoma have been inconclusive, with 3 of the 10 studies showing a positive association^11–13^ versus null results or even an inverse association in the others^14–20^. While all previous studies have used high-sensitivity CRP, the inconsistent results on this topic may be related to the influence of some studies with relatively smaller population sizes (n < 1,000)^14–16, 18, 20^.

Thus, this study was conducted in a relatively larger population of middle-aged and elderly Japanese. We quantified plasma concentrations of CRP using a highly sensitive method which enables the assessment of even low-grade inflammation, and evaluated the association between CRP level and the prevalence of colorectal adenoma.

## Materials and Methods

The Colorectal Adenoma Study in Tokyo was designed to investigate the role of dietary and lifestyle factors, including genetic susceptibility factors, in the early stage of colorectal carcinogenesis among healthy volunteer examinees of a colorectal cancer screening program.

### Study population

Between February 2004 and February 2005, a total of 3212 participants joined the cancer screening program of the Research Center for Cancer Prevention and Screening, National Cancer Center, Japan, and underwent colonoscopy examination. Inclusion criteria for the study have been detailed elsewhere^21–23^. Briefly, eligible participants were defined as men aged 50–79 years and women aged 40–79 years who had undergone total colonoscopy from the anus to cecum, with no history of colorectal adenoma, any type of cancer, ulcerative colitis, Crohn’s disease, familial adenomatous polyposis, carcinoid tumor, or colectomy. Since a small number of men under the age of 50 years joined the cancer screening program, their age cut-off was set at 50 years. 2234 examinees satisfied these conditions, of whom 526 men and 256 women had at least one adenoma by the pit pattern classification of colorectal lesions and were included as cases. Of the remaining 1452 examinees without adenoma, 482 men and 721 women were identified as eligible controls, after exclusion of those with inflammatory polyps, diverticulitis, submucosal tumor, and bowel tuberculosis or hyperplastic polyps. Due to the lower number of male controls than male cases, all male controls were included in the study, while 256 potential female controls were randomly selected by frequency matching with respect to age category (40–49, 50–54, 55–59, 60–64, and ≥65 years old) and screening period (first or second half) to cases. Thus, the study included a total of 782 cases and 738 controls. In this analysis, we further excluded 13 subjects with missing values for covariates and 88 nonsteroidal anti-inflammatory drug (NSAID) users. To limit the possibility of excessively high CRP levels due to acute inflammatory conditions such as infections or trauma, we also excluded 33 participants with the CRP value of 6 mg/L, meaning that they had CRP values above the upper detection limit. This left 729 cases (490 men and 239 women) and 657 controls (425 men and 232 women) for final analysis. All examinees provided written informed consent for the use of their screening data for medical research purposes. The study protocol, including all methods described in the present study, was approved by the institutional review board of the National Cancer Center (approval number G15–01, G16–03), in accordance to relevant ethical guidelines for medical research in Japan.

### Blood collection and laboratory assays

Fasting venous blood was collected before any screening examinations on the first day of screening. The collected blood samples were drawn into vacutainer tubes with ethylenediaminetetraacetic acid. The vacutainer tubes were centrifuged to obtain the plasma and buffy coat layers, and the specimens were stored at −80 °C until analysis.

Plasma C-reactive protein concentrations were measured using a latex-enhanced high-sensitivity assay on a BN II nephelometer (Dade Behring Marburg GmbH, Marburg, Germany) at a commercial laboratory (Mitsubishi Kagaku Bio-Clinical Laboratories, Inc., Tokyo, Japan). All laboratory personnel were blinded with respect to case or control status. The mean intra-assay coefficient of variation from the quality control sample in our previous study was 2.7% (n = 10)^24^.

### Self-administered questionnaire

Study subjects responded to a self-administered questionnaire before their screening examination which inquired about their demographics, lifestyle and socioeconomic characteristics, and personal and family medical history. Drinking status was initially categorized into three (past, current, or never). Among current drinkers, per-week alcohol consumption was calculated from the frequency of alcohol drinking and number of standard units consumed per occasion for five different alcoholic beverages (sake, shochu/awamori, beer, whisky, and wine). Smoking status was first categorized the same way into three (past, current, or never). Among ever smokers, lifetime exposure to cigarette smoking was measured by pack-years, in which one pack-year was defined as the smoking of 20 cigarettes every day for one year. The questionnaire also enquired about physical activities, including types of physical activity during working hours (sitting, standing, walking, or physically working) and the frequency of physical activity during leisure time (very light, light, moderate, or heavy). The total sum of physical activity combining occupational and leisure-time physical activity was expressed in metabolic equivalent task hours per day (METs-hour/day). BMI was calculated for each subject as the weight in kilograms divided by the height in meters squared (kg/m^2^). Participants also completed a 138-item food frequency questionnaire (FFQ), with pre-specified standard portion sizes and nine intake frequency categories for most food items.

### Statistical analysis

Case-control comparisons in mean, median, and proportions were tested with the *t*-test, Wilcoxon rank-sum test, and chi-square test, respectively. Given their skewed distribution, CRP values were natural logarithm-transformed. Dietary variables including total meat (g/day), fish (g/day), fiber (g/day), folate (μg/day), isoflavone (mg/day), fruits (g/day), and vegetables (g/day) were energy adjusted by the residual regression method^25^. In the association analysis, study subjects were divided into quartile categories based on the distribution of CRP values among the control group. Because men and women were selected by a different scheme, we used an unconditional logistic regression model to analyze both sexes together, and initially computed odds ratios (ORs) and 95% confidence intervals (CIs) with adjustment for sex, age (40–49, 50–54, 55–59, 60–64, ≥65 years), and screening period (model 1). Model 2 included further adjustment for family history of colorectal cancer, pack-years of smoking (never, ≤20, 21–40, >40 pack-years), drinking (never, past, <150, 150–299, ≥300 g/week), physical activity (METs-hour/day, quartile), and history of diabetes mellitus. Because obesity-induced inflammation might be an intermediate marker between obesity and colorectal neoplasia, we adjusted for BMI (<21.0, 21.0–22.9, 23.0–24.9, and ≥25.0 kg/m^2^) in a separate multivariate model. The linear trend test was performed by assigning ordinal values to the quartile categories of CRP and treating them as continuous variables in the regression model. Stratified analysis by sex and BMI (under versus above or equal to gender-specific median) was conducted and characteristics of adenomas (size and number) were also taken into account. Effect modification by sex and BMI on the associations with plasma CRP was assessed by the likelihood ratio test which compared the model with and without product terms of these variables. Two-sided *P* values < 0.05 were set to show statistical significance. All statistical analyses were done using SAS version 9.3 (SAS Institute, Inc., Cary, NC, USA).

## Results

### Baseline characteristics of study participants

Selected characteristics of cases and controls are summarized in Table 1. Compared to the controls, participants in the case group were slightly older, had a higher BMI, family colorectal cancer history and diabetes mellitus prevalence, and were more likely to be smokers. The case group also had a higher intake of total energy and total fiber, whereas they did not appear to differ from controls in terms of meat intake, physical activity, or alcohol drinking. The median CRP level was slightly higher in cases than in controls.

**Table 1:** Characteristics of all study subjects.

	Cases (n = 729)	Controls (n = 657)	*P* value
Men, n (%)	490 (67.2)	425 (64.7)	
Women, n (%)	239 (32.8)	232 (25.3)	
Continuous variables			
Age (years), mean (SD)	60.6 (6.1)	59.6 (5.9)	0.0037
BMI (kg/m^2^), mean (SD)	23.6(2.9)	23.0 (2.8)	0.0001
Physical activity (METs-hour/day), mean (SD)	37.1 (9.8)	36.5 (7.9)	0.18
Total energy (kcal/day), median (IQR)	1938 (1606–2359)	1855.0 (1558–2245)	0.0083
Total fiber (g/day)^b^, median (IQR)	12.3 (9.18–16.1)	12.9 (10.0–16.5)	0.012
Total meat (g/day)^b^, median (IQR)	45.3 (26.8–68.9)	43.6 (27.1–67.5)	0.35
CRP (mg/L), median (IQR)	0.4 (0.2–0.8)	0.3 (0.2–0.7)	0.0059
Categorical variables			
Smoking, n (%)			< 0.0001
Current smoker	146 (20.0)	69 (10.5)	
Past smoker	257 (35.3)	244 (37.1)	
Never smoker	326 (44.7)	344 (52.4)	
Drinking, n (%)			0.16
Current drinker	536 (73.5)	467 (71.1)	
Past drinker	36 (4.9)	24 (3.7)	
Never drinker	157 (21.5)	166 (25.3)	
Family CRC history, n (%)	121 (16.6)	79 (12.0)	0.016
Diabetes Mellitus, n (%)	68 (9.3)	40 (6.1)	0.025

^a^N, number of subjects; BMI, body mass index; METs, metabolic equivalents; CRC, colorectal cancer; SD, standard deviation; IQR, inter-quartile range.

^b^Energy-adjusted.

### Distribution of selected lifestyle and dietary factors by C-reactive protein quartile categories among controls

Variations in some of the lifestyle and dietary factors among controls are shown in Table 2. Participants with higher CRP levels tended to be slightly older than those with lower CRP levels. BMI also increased with increasing CRP level. In contrast, no apparent variation was seen with regard to physical activity, intake of total energy, total fiber, and energy-adjusted meat, smoking, alcohol drinking, family history of colorectal cancer, and prevalence of diabetes mellitus.

**Table 2:** Selected characteristics of controls according to C-reactive protein level.

	Quartile of C-reactive protein (mg/L)				*P*_trend_
	Lowest	Second	Third	Highest	
Men, n (%)	87 (20.5)	108 (25.4)	118 (27.8)	112 (26.4)	
Women, n (%)	77 (33.2)	50 (21.5)	51 (22.0)	54 (23.3)	
Continuous variables					
Age (years), median (IQR)	58 (54.5–62.5)	60 (56.0–65.0)	60 (56.0–64.0)	61 (56.0–64.0)	0.081
BMI (kg/m^2^), median (IQR)	21.6 (20.1–23.4)	22.9 (20.9–24.2)	23.3 (21.3–25.0)	23.9 (22.2–25.9)	<0.0001
Physical activity (METs-hour/day), median (IQR)	35.5 (31.5–39.7)	34.3 (31.4–38.8)	33.9 (30.8–38.1)	34.1 (31.9–38.3)	0.40
Total energy (kcal/day), median (IQR)	1870 (1664–2240)	1834 (1553–2269)	1883 (1522–2236)	1853 (1482–2227)	0.60
Total fiber (g/day)^b^, median (IQR)	13.3 (10.4–17.0)	12.7 (10.0–16.5)	12.9 (9.6–17.0)	12.8 (10.0–16.4)	0.80
Total meat (g/day)^b^, median (IQR)	44.1 (29.0–69.6)	43.2 (28.6–67.0)	41.6 (25.0–61.2)	46.0 (28.2–71.7)	0.47
CRP (mg/L), median (IQR)	0.1 (0.08–0.1)	0.3 (0.2–0.3)	0.5 (0.4–0.6)	1.2 (0.9–1.8)	<0.0001
Categorical variables					
Smoking, n (%)					0.24
Current smoker	13 (7.9)	17 (10.8)	16 (9.5)	23 (13.9)	
Past smoker	52 (31.7)	59 (37.3)	69 (40.8)	64 (38.6)	
Never smoker	99 (60.4)	82 (51.9)	84 (49.7)	79 (47.6)	
Drinking, n (%)					0.89
Current drinker	118 (72.0)	114 (72.2)	117 (69.2)	118 (71.1)	
Past drinker	4 (2.4)	8 (5.1)	6 (3.6)	6 (3.6)	
Never drinker	42 (25.6)	36 (22.8)	46 (27.2)	42 (25.3)	
Family CRC history, n (%)	20 (12.2)	21 (13.3)	21 (12.4)	17 (10.2)	0.86
Diabetes Mellitus, n (%)	11 (6.7)	9 (5.7)	8 (4.7)	12 (7.2)	0.78

^a^n, number of subjects; BMI, body mass index; METs, metabolic equivalents; CRC, colorectal cancer; SD, standard deviation; IQR, inter-quartile range.

^b^Energy-adjusted.

### Association of C-reactive protein with colorectal adenoma

Plasma CRP concentrations was positively associated with the prevalence of colorectal adenoma, albeit in a nonlinear manner (Table 3). The OR of colorectal adenoma for the highest compared to the lowest quartile of plasma CRP in the overall population was 1.30 (95% CI 0.94–1.79), with *P* trend = 0.031. When this analysis was stratified by gender, a similar pattern of non-linear positive association was also observed among men, whereas an attenuated pattern was seen in women; the corresponding OR was 1.41 (95% CI 0.93–2.13), with *P* trend = 0.018, in men and 0.99 (95% CI 0.58–1.70), with *P* trend = 0.62, in women. Effect modification by sex was not statistically significant (*P* interaction = 0.281). When stratified by BMI, the association between plasma CRP and colorectal adenoma did not differ according to BMI category (*P* interaction = 0.794).

**Table 3:** Association of C-reactive protein with colorectal adenoma, by gender and BMI.

	Quartile of C-reactive protein (mg/L)				*P*_trend_
	Lowest	Second	Third	Highest	
All subjects					
Cases/controls	138/164	159/158	228/169	204/166	
OR (95% CI)^a^	1.0 (Reference)	1.17 (0.85–1.61)	1.58 (1.16–2.14)	1.44 (1.05–1.96)	0.0097
OR (95% CI)^b^	1.0 (Reference)	1.12 (0.81–1.55)	1.51 (1.11–2.06)	1.30 (0.94–1.79)	0.031
OR (95% CI)^c^	1.0 (Reference)	1.06 (0.76–1.47)	1.35 (0.98–1.86)	1.12 (0.80–1.56)	0.25
Stratified by Gender*					
Men					
Cases/controls	73/87	101/108	167/118	149/112	
OR (95% CI)^a^	1.0 (Reference)	1.11 (0.73–1.68)	1.70 (1.14–2.52)	1.61 (1.08–2.40)	0.0056
OR (95% CI)^b^	1.0 (Reference)	1.04 (0.68–1.60)	1.63 (1.09–2.43)	1.41 (0.93–2.13)	0.018
OR (95% CI)^c^	1.0 (Reference)	0.97 (0.63–1.50)	1.44 (0.96–2.18)	1.22 (0.80–1.88)	0.11
Women					
Cases/controls	65/77	58/50	61/51	55/54	
OR (95% CI)^a^	1.0 (Reference)	1.39 (0.84–2.31)	1.44 (0.87–2.37)	1.22 (0.73–2.04)	0.37
OR (95% CI)^b^	1.0 (Reference)	1.17 (0.68–1.99)	1.30 (0.77–2.20)	0.99 (0.58–1.70)	0.62
OR (95% CI)^c^	1.0 (Reference)	1.13 (0.66–1.93)	1.16 (0.67–2.00)	0.83 (0.47–1.49)	0.95
Stratified by BMI**					
BMI ≥ median					
Cases/controls	49/46	77/60	139/85	137/103	
OR (95% CI)^a^	1.0 (Reference)	1.08 (0.63–1.85)	1.40 (0.85–2.31)	1.11 (0.68–1.81)	0.69
OR (95% CI)^b^	1.0 (Reference)	1.01 (0.58–1.77)	1.34 (0.80–2.26)	0.99 (0.59–1.66)	0.90
BMI < median					
Cases/controls	89/118	82/98	89/84	67/63	
OR (95% CI)^a^	1.0 (Reference)	1.14 (0.76–1.72)	1.43 (0.94–2.16)	1.45 (0.93–2.28)	0.057
OR (95% CI)^b^	1.0 (Reference)	1.08 (0.71–1.64)	1.27 (0.83–1.94)	1.22 (0.75–1.96)	0.18

^a^Adjusted for age (40–49, 50–54, 55–59, 60–64, ≥65 years), sex (except when stratified by gender), and screening period.

^b^Further adjusted for family colorectal cancer history, pack-years of smoking (never, ≤20, 21–40, >40 pack-years), alcohol drinking (never, past, <150, 150–299, ≥300 g per week), physical activity (METs-hour/day, quartile), and history of diabetes mellitus.

^c^Further adjusted for body mass index (BMI) (<21.0, 21.0–22.9, 23.0–24.9, and ≥25.0 kg/m^2^).

^*^Interaction for sex was not significant (P = 0.281).

**Interaction for BMI was not significant (P = 0.794).

The observed positive association of plasma CRP level with colorectal adenoma overall as well as in male subjects, however, lost statistical significance when further adjusted for BMI. The OR of adenoma for the highest compared to the lowest quartile of plasma CRP was 1.12 (95% CI 0.80–1.56), with *P* trend = 0.25, in overall subjects and 1.22 (95% CI 0.80–1.88), with *P* trend = 0.11, in men after adjusting for BMI. Additional adjustment by energy-adjusted dietary factors, including total meat, fiber, folate, fruits, vegetables, isoflavone, and fish, did not substantially change the result from the BMI-adjusted model (results not shown).

### Association of C-reactive protein with colorectal adenoma by size and number of adenomas

We further examined the association between plasma CRP level and colorectal adenoma by the size and number of adenomas (Table 4). Of 729 adenoma cases, 326 (44.7%) had a larger adenoma (≥5 mm) and 314 (43.1%) had multiple adenomas (≥2). Circulating CRP levels appeared to be positively associated with the prevalence of colorectal adenoma in those with a larger adenoma (≥5 mm) but not in those with smaller adenoma (<5mm), and also in those with multiple adenomas (≥2) but not in those with a single adenoma. When stratified by gender, the above positive associations with larger and multiple adenomas were clearly observed in men but became vague in women (results not shown).

**Table 4:** Association of C-reactive protein with colorectal adenoma by size and number.

	Quartile of C-reactive protein (mg/L)				*P*_trend_
	Lowest	Second	Third	Highest	
Controls	164	158	169	166	
Size					
≥5mm					
Cases	56	72	101	97	
OR (95% CI)^a^	1.0 (Reference)	1.26 (0.83–1.91)	1.66 (1.11–2.48)	1.64 (1.09–2.45)	0.012
OR (95% CI)^b^	1.0 (Reference)	1.20 (0.78–1.84)	1.59 (1.06–2.39)	1.48 (0.98–2.25)	0.031
OR (95% CI)^c^	1.0 (Reference)	1.15 (0.74–1.77)	1.47 (0.97–2.23)	1.33 (0.86–2.05)	0.14
<5mm					
Cases	82	87	127	107	
OR (95% CI)^a^	1.0 (Reference)	1.10 (0.76–1.60)	1.52 (1.06–2.17)	1.31 (0.91–1.88)	0.074
OR (95% CI)^b^	1.0 (Reference)	1.06 (0.72–1.55)	1.50 (1.04–2.15)	1.20 (0.83–1.76)	0.12
OR (95% CI)^c^	1.0 (Reference)	0.99 (0.67–1.45)	1.32 (0.91–1.92)	1.02 (0.69–1.50)	0.53
Number					
≥2					
Cases	43	75	98	98	
OR (95% CI)^a^	1.0 (Reference)	1.65 (1.06–2.57)	2.02 (1.31–2.09)	2.04 (1.33–3.14)	0.0013
OR (95% CI)^b^	1.0 (Reference)	1.58 (1.00–2.48)	1.93 (1.25–3.00)	1.84 (1.18–2.87)	0.0032
OR (95% CI)^c^	1.0 (Reference)	1.49 (0.94–2.35)	1.71 (1.09–2.68)	1.53 (0.96–2.43)	0.048
1					
Cases	95	84	130	106	
OR (95% CI)^a^	1.0 (Reference)	0.96 (0.66–1.38)	1.43 (1.01–2.03)	1.18 (0.82–1.68)	0.16
OR (95% CI)^b^	1.0 (Reference)	0.91 (0.63–1.33)	1.37 (0.96–1.96)	1.08 (0.74–1.56)	0.27
OR (95% CI)^c^	1.0 (Reference)	0.86 (0.59–1.27)	1.26 (0.87–1.81)	0.96 (0.65–1.40)	0.68

^a^Adjusted for age (40–49, 50–54, 55–59, 60–64, ≥65 years), sex, and screening period.

^b^Further adjusted for family colorectal cancer history, pack-years of smoking (never,≤20, 21–40, >40 pack-years), alcohol drinking (never, past, <150, 150–299, ≥300 g per week), physical activity (METs-hour/day, quartile), and history of diabetes mellitus.

^c^Further adjusted for body mass index (BMI) (<21.0, 21.0–22.9, 23.0–24.9, and ≥25.0 kg/m^2^).

## Discussion

In this study, we observed a positive association between plasma CRP concentration and the prevalence of colorectal adenoma. Given that CRP is a preferable biomarker of systemic inflammation and that a highly sensitive method can evaluate low-grade inflammation, our present finding of a positive association between higher CRP levels and an increased prevalence of adenoma supports the hypothesis that the development/growth of colorectal neoplasia likely involves a systemic, low-grade inflammatory state.

Of importance, the present study showed a clear influence of BMI on the association between plasma concentrations of CRP and colorectal adenoma. An increase in CRP levels was associated with an increased risk of colorectal adenoma only before BMI was taken into account, suggesting that the observed association can be ascribed to obesity-related inflammation. Indeed, inflammatory cytokines derived from adipose tissues, specifically IL-6 and TNF-α, have been well characterized to activate oncogenic transcription pathways of many types of cancer, including colorectal cancer^8, 26–28^. After binding to its receptor, IL-6 triggers both the nuclear factor κB (NF-κB) cascade and STAT3 pathway, which are responsible for cell survival/tumor initiation and cell proliferation/tumor growth downstream, respectively^28, 29^. In addition, TNF-α is involved in starting the NF-κB and activator protein-1 (AP-1) signaling pathways to promote cell proliferation and survival^28, 30^. Thus, the low-grade inflammation established by obesity may play an important role in colorectal carcinogenesis, including its early stages.

In our present observations, the CRP-adenoma association was more evident with larger and multiple adenomas than with smaller and single adenomas. Colorectal adenoma is widely considered to be a precursor to colorectal cancer, and the majority of colorectal cancers are known to arise from colorectal adenoma^31, 32^. More importantly, adenoma of an increased size and number are at a greatly elevated risk of malignant conversion. These findings underscore the importance of colorectal adenoma status in relation to colorectal cancer^33^. The Tennessee Colorectal Polyp Study (TCPS) in the US identified a positive association between circulating CRP and colorectal adenoma, but notably only for advanced and multiple adenomas^13^. Two other studies provide further evidence for the association between circulating CRP and advanced and synchronous adenoma^11, 12^: Otake *et al*. found that the OR of circulating CRP associated with an adenoma size equal to or larger than 5 mm was more than 2^12^, while Chiu *et al*. observed a positive association only in the presence of larger or multiple adenomas, highlighting the impact of size and number on the CRP-adenoma association. Interestingly, this positive association was retained in concurrent advanced and synchronous adenoma cases, which showed the further elevation of OR^11^.

Although no significant interaction was observed, our findings showed a possible sex difference, in which the association between plasma CRP level and colorectal adenoma was relatively more apparent in men. A similar gender difference has been consistently observed in many prospective studies of obesity and colorectal cancer as well as their meta-analyses^1, 5, 34–36^, but less consistently in studies of colorectal adenoma^2, 37^. Indeed, a meta-analysis on the association between BMI and colorectal cancer by Wang *et al*. found that colorectal cancer was the only one of a number of different cancer types to show a significant gender difference^35^. In the meantime, a Taiwanese cross-sectional study of circulating CRP and colorectal adenoma showed a non-linear positive increase in adenoma OR only in male subjects when stratified by gender^11^, which is consistent with our present findings. However, the gender difference in our study and in the Taiwanese study should be interpreted with caution as their sample sizes in women were nearly half those in men, and the resulting low statistical power may have obscured the true association in women. Evidence for this possible gender difference in colorectal adenoma in relation to circulating CRP remains limited, and further investigation is warranted.

A strength of our study is that all participants underwent total colonoscopy, reducing the possibility of misclassification of case and control status. Other strengths include our large sample size and adjustment for known and potential risk factors. Among limitations, one is the cross-sectional design of the study, which prevents any assignment of causality. Nevertheless, colorectal adenoma is unlikely to affect adiposity, as the underlying studies unanimously show the influence of BMI on low-grade systemic inflammation and adenoma, but not vice versa^4, 38, 39^. Colorectal adenoma cases in our study were not pathologically verified, and single measurement of plasma CRP may not have completely represented random variation within the subjects. Incorporating additional steps to reduce this possibility could have led to a further increase in the reliability of our results. Finally, as previously mentioned^23^, it is important to note that the present study was based on prevalent cases instead of incident cases, which suggests that the ORs in this study cannot necessarily be applied to infer the risk of adenoma “development”. Rather, they can only imply the risk of “having” an adenoma at a given time point, and should thus be interpreted carefully.

In summary, this study in a middle-aged and elderly Japanese population showed a positive association between plasma CRP level and colorectal adenoma, notably in larger and multiple adenomas. However, the positive association was attenuated after adjustment for BMI, which likely suggests that plasma CRP level was largely correlated with BMI. Thus, our results may suggest the importance of an obesity-related systemic, low-grade inflammatory state in relation to colorectal carcinogenesis, at least in its early stages. Pathological evaluation of adenomas and measurement of circulating inflammatory cytokines can provide further insight into the molecular mechanisms of obesity in the etiology of colorectal neoplasia.
